# Computer-Aided Construction and Evaluation of Poly-L-Lysine/Hyodeoxycholic Acid Nanoparticles for Hemorrhage and Infection Therapy

**DOI:** 10.3390/pharmaceutics17010007

**Published:** 2024-12-24

**Authors:** Qin Qin, Wenxing Wu, Ling Che, Xing Zhou, Diedie Wu, Xiaohui Li, Yumin Yang, Jie Lou

**Affiliations:** 1 School of Pharmacy and Bioengineering, Chongqing University of Technology, Chongqing 400054, China; qinqin@stu.cqut.edu.cn (Q.Q.); 18223447972@163.com (D.W.); 2 Department of Pharmaceutics, College of Pharmacy, Army Medical University, Chongqing 400038, China; w242900@hotmail.com (W.W.); lpsh008@aliyun.com (X.L.); 3 Department of Pharmacy, Medical Supplies Center of PLA General Hospital, Beijing 100853, China; cheling309@126.com; 4 Yunnan Key Laboratory of Stem Cell and Regenerative Medicine, Science and Technology Achievement Incubation Center, Kunming Medical University, Kunming 650500, China; diszhou@126.com

**Keywords:** computer simulation, hemostasis, antibacterial, poly-L-lysine, cholic acid derivatives, drug delivery

## Abstract

**Background:** Traumatic hemorrhage and infection are major causes of mortality in wounds caused by battlefield injuries, hospital procedures, and traffic accidents. Developing a multifunctional nano-drug capable of simultaneously controlling bleeding, preventing infection, and promoting wound healing is critical. This study aimed to design and evaluate a nanoparticle-based solution to address these challenges effectively. **Methods:** Using a one-pot assembly approach, we prepared a series of nanoparticles composed of poly-L-lysine and hyodeoxycholic acid (PLL-HDCA NPs). Theoretical simulations and experimental studies were combined to optimize their structure and functionality. In vitro platelet aggregation, antibacterial assays, cytotoxicity tests, and hemolysis evaluations were performed. In vivo efficacy was assessed in various hemorrhage models, a full-thickness skin defect model, and a skin irritation test. **Results:** PLL-HDCA NPs demonstrated effective induction of platelet aggregation and significantly reduced bleeding time and blood loss in mouse models, including tail vein, femoral vein, artery, and liver bleeding. Antibacterial assays revealed strong activity against *E. coli* and *S. aureus*. Wound healing studies showed that PLL-HDCA NPs promoted tissue repair in a full-thickness skin defect model. Cytotoxicity and hemolysis tests indicated minimal impact on human cells and significantly reduced hemolysis rates compared to PLL alone. Skin irritation tests confirmed the safety of PLL-HDCA NPs for external application. **Conclusions:** PLL-HDCA NPs represent a safe, efficient, and multifunctional nano-drug suitable for topical applications to control bleeding, combat infection, and facilitate wound healing, making them promising candidates for use in battlefield and hospital settings.

## 1. Introduction

Hemostasis and infection prevention are crucial challenges in trauma management [1]. Materials with both hemostatic and antibacterial properties are essential to mitigate hemorrhagic shock and post-traumatic infections resulting from blood loss and bacterial contamination [1,2]. While controlling bleeding is paramount, the prevention of wound infections is equally crucial, as infections can significantly worsen wound prognosis and increase the burden of wound care. Historically, individuals with severe injuries often required high doses of antibiotics, either orally or intravenously, to prevent infections [3]. Currently, commercially available hemostatic materials like QuikClot (zeolite and kaolin) [4], Celox (chitosan), instant yarn (regenerated oxidized cellulose) [5] et al. are widely used. However, these materials primarily address bleeding and lack antibacterial properties. Therefore, there is a pressing need to develop advanced hemostatic materials capable of providing both rapid hemostasis and effective infection prevention.

Computer-aided drug design (CADD) is a method of rationally designing new structural compounds by simulating drug-target interactions based on the principles of computational chemistry. This approach has become a routine and cost-effective method for drug discovery, accelerating the development of novel concepts to address persistent diseases. Recent advancements in CADD techniques, including molecular docking and molecular dynamics simulations, have enhanced the prediction of intermolecular interactions and are widely used in drug design [6]. In this project, we employed computer-aided simulation and virtual screening to explore the processes and mechanisms underlying the formation of nanoparticles composed of two components.

Previous research introduced a strategy for developing a novel drug delivery system. Studies have demonstrated that positively charged nanoparticles can bind to negatively charged fibrinogen at a pH of approximately 5.5 [7]. This electrostatic interaction induces platelet aggregation, triggering coagulation and positioning these nanoparticles as potential nanohemostatic agents. To further explore this potential, we utilized poly-L-lysine (PLL), an FDA-approved biodegradable material known for its numerous advantageous properties, including high drug-loading capacity, biocompatibility, targeted release, and controlled delivery. Moreover, PLL nanocarriers can cross the blood–brain barrier and reticuloendothelial tissue system, exhibit broad-spectrum antibacterial activity, and promote platelet aggregation [8,9]. However, PLL’s strong cationic charge often results in adsorption to the surface of red blood cells, leading to hemolytic toxicity that limits its clinical application. Consequently, PLL is primarily employed as a carrier for drug delivery or food antiseptics [10]. Despite this limitation, PLL’s abundant amino groups enable efficient conjugation and modification [11,12,13]. Additionally, PLL can self-assemble through electrostatic interactions, forming nanoparticles with anion-containing drugs. This study proposes an innovative approach where PLL serves as both a carrier and a multifunctional antibacterial and hemostatic agent, creating nanoparticles with high drug-loading capacity and minimized safety risks.

Cholic acid derivatives are organic acids characterized by a steroid backbone and an anionic carboxyl group [14]. Some derivatives have demonstrated antibacterial effects, while others exhibit pharmacological properties such as antispasmodic, stomachic, and biocompatibility effects [15]. However, their poor water solubility poses a significant challenge for therapeutic applications due to slow dissolution rates and low oral bioavailability [16]. Nano-drug delivery systems present a promising solution to enhance the clinical potential of cholic acid derivatives. Given the hydrophobic nature of cholic acid derivatives, which contain anionic carboxyl groups, and the hydrophilic cationic amino groups of poly-L-lysine (PLL) [17], we hypothesize that cholic acid derivatives can self-assemble with PLL through hydrophobic van der Waals forces and electrostatic interactions, resulting in nanomedicines with both hemostatic and antibacterial properties.

**Figure 1:**
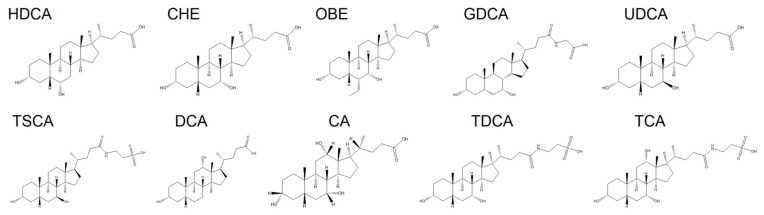
Molecular structures of cholic acid derivatives.

Based on these insights, ten cholic acid derivatives were selected for investigation, including chenodeoxycholic acid (CHE), cholic acid (CA), deoxycholic acid (DCA), hyodeoxycholic acid (HDCA), glycochenodeoxycholic acid (GDCA), obeticholic acid (OBE), taurochenodeoxycholic acid (TDCA), taurocholic acid (TCA), tauroursodeoxycholic acid (TSCA), and ursodeoxycholic acid (UDCA) (Figure 1). These derivatives were formulated into nanoparticles using a nanoparticle preparation method with poly-L-lysine (PLL). Computer simulations were then used to validate the experimental results, leveraging their ability to accurately predict intermolecular interactions between hydrophilic polymers and hydrophobic drugs. In vitro studies were conducted to investigate the interactions between cholic acid derivatives and PLL, characterize the assembled nanoparticles, and evaluate their effects on platelet aggregation. Furthermore, the hemostatic efficacy of the nanoparticles was assessed in various hemorrhage models following topical application. Their antibacterial activity and wound healing capabilities were also examined.

## 2. Materials and Methods

### 2.1. Materials and Animals

Cholic acid derivatives and PLL (M.W.15,000–30,000 Da) were obtained from Sigma-Aldrich (St. Louis, MO, USA). Penicillin, streptomycin, fetal bovine serum (FBS), and Dulbecco’s modified eagle medium (DMEM) were purchased from HyClone (Waltham, MA, USA). DMSO was purchased from Sinopharm Chemical Reagent, China. All the other reagents are commercially available and used as received. *E. coli* and *S. aureus* were kindly provided by researchers from Sichuan University. Kunming mice were supported by the animal center of Chongqing University of Technology. Further details on our ethics and experimental platform can be found in the Ethical Statement section located at the end of this article.

### 2.2. Preparation and Characterization of Cholic Acid Derivatives-PLL Nanoparticles

The cholic acid structure was used as the base pharmacophore, and ten derivatives (CHE, CA, DCA, HDCA, GDCA, OBE, TDCA, TCA, TSCA, UDCA) were identified from the Drug Bank database (https://www.drugbank.ca/, accessed on 23 September 2019). These derivatives served as building blocks for the preparation of cholic acid derivative-PLL nanoparticles, synthesized using the nanoprecipitation method. Specifically, cholic acid derivatives and PLL (molecular weight: 15,000–30,000 Da) were dissolved in DMSO at a 1:1 mass ratio, maintaining a polymer concentration of 5 mg/mL in all cases. The resulting solution was placed in a 3 kDa dialysis bag and dialyzed against deionized water for 8 h at a rotational speed of 300 rpm. The outer aqueous phase was replaced every 30 min for the first 3 h (6 times) and hourly for the remaining 5 h. The nanoparticles were collected via centrifugation and used for subsequent analyses without further processing.

Particle size distribution, polymer dispersity index (PDI), and zeta potential were measured using laser granulometry (Nano-ZS90, Malvern Instruments; Brookhaven Instruments, New York, NY, USA) at room temperature. Particle morphology was observed using transmission electron microscopy (TEM, S-3400 n II electron microscope; New York, NY, USA). Stability testing for particle size and zeta potential was performed on days 0, 1, 2, 3, 4, 5, 6, 7, 30, and 60, using the Nano-ZS90. Fourier-transform infrared (FTIR) spectroscopy (PerkinElmer, Rodgau, Germany) in reflectance mode was utilized to investigate functional groups in cholic acid derivatives, poly-L-lysine (PLL), cholic acid derivative-PLL mixtures, and cholic acid derivative-PLL nanoparticles within a wavelength range of 500–4000 cm^−1^. The phase composition of the products was analyzed using a Panalytical Empyrean diffractometer (Malvern Panalytical Ltd., Malvern, UK) with Cu-Kα radiation (λ = 1.5406 Å) operated at 45 kV and 40 mA. X-ray diffraction (XRD) patterns were recorded over a 2θ range of 5–90° at a scanning rate of 5°/min. Drug loading and encapsulation efficiency of PLL-HDCA nanoparticles (NPs) were determined using high-performance liquid chromatography (HPLC). Samples were dissolved in methanol, and the mobile phase consisted of acetonitrile with 0.1% phosphoric acid. Detection was carried out at a wavelength of 192 nm. The HDCA loading content was calculated using the following equation:

(1) $$\mathrm{Drug}\;\mathrm{loading}\;\mathrm{content}=\frac{mHDCA}{mPLL-HDCANPs}\times 100\%$$

(2) $$\mathrm{Encapsulation}\;\mathrm{efficiency}=\frac{mHDCA}{\;\;\;mTOTAL}\times 100\%$$

where *mHDCA* represents the mass of HDCA, *mPLL-HDCA NPs* represents the mass of PLL-HDCA NPs, and *mTOTAL* represents the total mass of drug.

### 2.3. Computer Simulation

**Figure 2:**
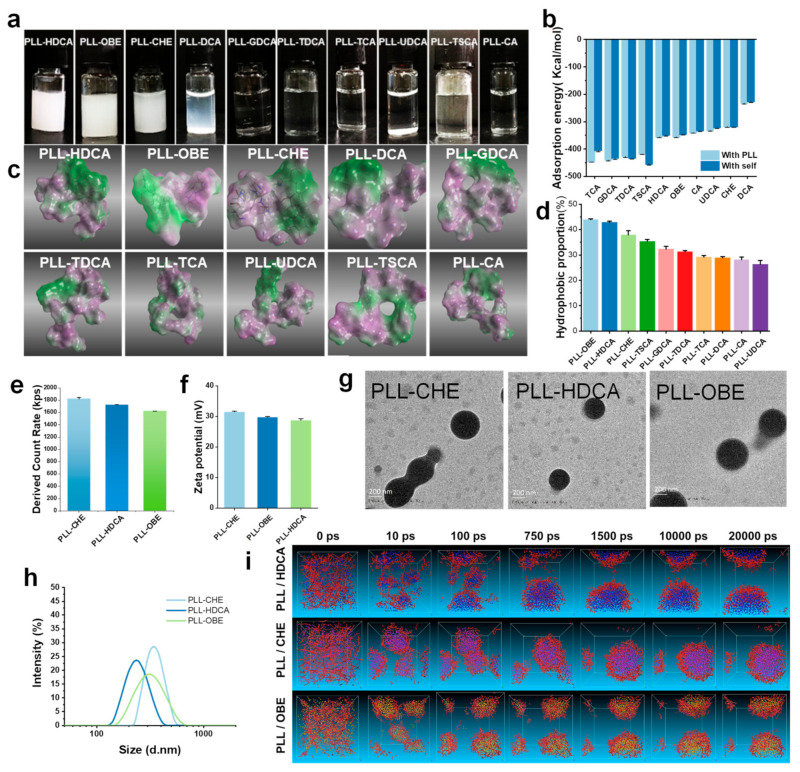
The characterization and computer simulation of PLL-cholic acid derivatives NPs. (**a**) Visual inspection of PLL-cholic acid derivatives complexes. (**b**) Adsorption energies between PLL and cholic acid derivatives. (**c**) The hydrophilic and lipophilic surfaces of PLL-cholic acid derivatives complexes after MD simulation. The green area represents hydrophobic, and the purple area represents hydrophilic. (**d**) The proportion of hydrophobic regions was statistically analyzed on the amphiphilic map of PLL-cholic acid derivative complexes. (**e**) Count rate distribution. (**f**) Zeta-potential distribution. (**g**) The TEM image of PLL-CHE\HDCA\OBE NPs. (**h**) The particle size distribution. (**i**) The dynamic mesostructure of PLL-cholic acid derivatives complexes during 20 ns DPD simulation. PLL beads were represented as red colors. HDCA, CHE, and OBE beads were, respectively, represented as blue, purple and yellow colors. Data are mean ± SD (*n* = 3).

The structures of the cholic acid derivatives and PLL were obtained from the DrugBank database (https://www.drugbank.ca/, accessed on 8 October 2019). The PLL chain was constructed by a head-to-tail connection with 20 structural units on Materials Studio (MS) 2017 software (Accelrys Inc, kindly provided by Analytical & Testing Center of Sichuan University). Geometry optimization was performed using the COMPASS force field, followed by a molecular docking to calculate adsorption energy values (Figure 2b). Mixing energy values were subsequently calculated using the Blends module. To evaluate the stability of complex molecules, a molecular dynamics (MD) simulation was conducted for 10,000 ps using the Dynamics task of the Forcite module. Final conformations were processed in the Molecular Operating Environment (MOE, version MOE 2015.10) software (Chemical Computing Group), and the percentage area of the hydrophobic region was analyzed using the Image J software (Image J 1.51j8; NIH). For coarse-grained modeling of PLL and cholic acid derivative assembly, dissipative particle dynamics (DPD) simulation was employed. Repulsion parameter (a_ij_) data (Table S1) for DPD simulation were generated using the Blends module, following the formula a_ij(ρ=3)_ = 78 + 3.50 × Chi. After establishing a mesoscale model with a Water:PLL:Cholic acid derivatives ratio of 1:8:1 (Figure S3), a force field was generated through the DPD method, using a length scale of 6.46 Å and a mass scale of 54 amu. A DPD simulation was then performed for 20 ns. In AutoDock 4.2, the optimized molecular conformation of HDCA\CHE\OBE was imported as a ligand, while the optimized molecular conformation of PLL was imported as a macromolecule. The grid box dimensions were set to 126 Å × 126 Å × 126 Å, with each small grid point spaced at a distance of 0.0375 nm. The calculations employed the Lamarckian genetic algorithm, simulated annealing algorithm, and local search algorithm, with all other parameters maintained at their default settings.

### 2.4. In Vitro Pro-Coagulant Assay of PLL-HDCA NPs

The in vitro hemostasis ability of the PLL-HDCA NPs was evaluated by measuring whole blood coagulation time and clotting area. Whole blood was collected from mice using 3.8% sodium citrate (Sigma-Aldrich) as an anticoagulant. PLL-HDCA NPs, PLL, and HDCA were each dissolved in normal saline. Subsequently, 40 μL of whole blood, 10 μL 0.1 M CaCl_2_ and 20 μL drug solution (1 mg/mL) were sequentially added to a 96-well. Every 60 s, 200 μL of saline was gently added to rinse unbound blood without disturbing the clots. Coagulation time was recorded, and clot areas were measured using Image J software. The clot area was expressed as a proportion of the total area.

To investigate the mechanism of nanoparticle-induced coagulation, the effect of the nanoparticles on platelet aggregation was assessed using electron microscopy. Whole blood was collected from mice with 3.8% sodium citrate as an anticoagulant. Platelet-rich plasma (PRP) was obtained by centrifugation at 900 rpm for 20 min. A mixture of 40 μL of PRP, 20 μL of 0.1 M CaCl_2_, and 20 μL of drug solution (1 mg/mL) was incubated for 10 min, followed by staining with Wright’s staining solution and rinsing with distilled water. Platelet morphology was observed under an electron microscope at 40× magnification.

### 2.5. In Vivo Hemostatic Performance of PLL-HDCA NPs

The in vivo hemostatic performance of the PLL-HDCA NPs was evaluated with various hemorrhage models, including tail vein, femoral vein, femoral artery, and liver models.

Tail Vein Hemorrhage Model: Kunming mice (5–6 weeks-old, male) were randomly assigned into four groups (saline, PLL, HDCA, and PLL-HDCA NPs). Syringe needles were inserted into the tail vein at a 30° angle and then slowly withdrawn, followed by topical administration of the hemostatic treatments (1 mg/mL) for each group. Bleeding time and blood loss were measured.

Femoral Vein and Artery Hemorrhage Models: Kunming mice (5–6 weeks old, male) were randomly assigned to the same four groups as described above. The mice were anesthetized and secured on a surgical corkboard. Syringe needles were inserted into the femoral vein or artery at a 45° angle and then slowly removed. Hemostatic treatments (1 mg/mL) were topically applied, and bleeding time and blood loss were recorded.

Liver Hemorrhage Model: Kunming mice (5–6 weeks-old, male) were randomly divided into four groups as described above. After anesthesia, the mice were fixed on a surgical corkboard, and the liver was exposed through an abdominal incision. The left lobe of the liver was gently placed on pre-weighed filter paper. A sterile scalpel was used to create a wound 2 mm in depth and 5 mm in width on the left lobe, followed by topical application of the hemostatic agents. A pre-weighed filter paper was immediately applied to the bleeding site. Bleeding time and blood loss were recorded.

### 2.6. Antibacterial Capability of PLL-HDCA NPs

The antibacterial properties of PLL-HDCA nanoparticles (NPs) were evaluated using Gram-positive *Staphylococcus aureus* (*S. aureus*, ATCC 6538) and Gram-negative *Escherichia coli* (*E. coli*, ATCC 25922). Both bacterial strains were cultured on LB agar plates and exposed to various drug concentrations, with PBS serving as the control group. The antibacterial activity against *S. aureus* and *E. coli* was determined by the plate-counting method. In a 96-well plate, 100 μL of *S. aureus* suspension (1 × 10^5^ CFU/mL was mixed with equal volumes of PBS, PLL, HDCA, or PLL-HDCA NPs and incubated at 37 °C for 24 h. Subsequently, 100 μL bacterial broth was transferred for plate coating and incubated at 37 °C for 24 h, followed by colony counting using a G6 automatic colony counter (Shineso; Hangzhou Xunshu Technology Co., Ltd., Hangzhou, China).

Bacterial morphology was analyzed using scanning electron microscopy (SEM, Inspect F50; FEI, Company, Hillsboro, OR, USA). *E. coli* was treated with HDCA or PLL-HDCA NPs at a concentration corresponding to PLL (0.375 mg/mL), while *S. aureus* was treated at 0.1875 mg/mL. After centrifugation, the bacteria were fixed with 2.5% glutaraldehyde for 4 h at room temperature. Fixed bacteria were washed with sterile saline, dehydrated using a graded ethanol series (30%, 50%, 70%, 80%, 90%, 95%, and 100%) for 10 min at each step, and air-dried overnight. Before imaging, bacteria were sputter-coated with platinum and visualized under SEM.

Cell membrane integrity was analyzed as described in with slight modifications [18]. Briefly, *S. aureus* and *E. coli* cells were incubated with propidium iodide (PI) (10 μg/mL; Sigma-Aldrich, Castle Hill, NSW, Australia) for 30 min at 4 °C in the dark. Membrane integrity was assessed using flow cytometry (BD FACSAria III).

### 2.7. Ability of PLL-HDCA NPs to Promote Wound Healing

The wound-healing potential of PLL-HDCA NPs was evaluated using a skin injury model. Kunming mice (30–40 g) were randomly assigned to four groups: Saline, HDCA, PLL, and PLL-HDCA NPs (*n* = 3 per group). After anesthesia, the backs of each mouse were shaved, and two full-thickness wounds (6 mm in diameter) were created on either side of the midline using a biopsy punch. The wounds were disinfected with povidone iodine, and the respective treatments were topically applied on days 1, 3, 5, 7, 9, 10, 11, 13, and 15 post-surgery. On day 7, wound tissues were collected and fixed in 4% paraformaldehyde for histological analysis. Wound size was measured and photographed on days 0, 3, 7, 11, and 15, and ImageJ software was used to calculate the wound area and healing rate. Histopathological sections of wound tissues were analyzed by hematoxylin and eosin (H&E) staining and imaged using a microscope (NIKON CI-S, Tokyo, Japan). The wound healing rate was calculated using the formula:

Healing rate (%) = (A_0_ − A_n_)/A_0_ × 100 (3)

where A_0_ represents the initial wound size on day 0, and A_n_ represents the wound size on days 3, 7, 11, and 15.

### 2.8. Biocompatibility of PLL-HDCA NPs

The biocompatibility of PLL-HDCA NPs was assessed through cytotoxicity assays, hemolysis assays, and skin irritation tests. Cytotoxicity was evaluated using human skin fibroblasts (HSF cells) in a Cell Counting Kit-8 (CCK-8) assay. HSF cells were cultured in DMEM supplemented with 10% FBS (containing 1% penicillin-streptomycin) at a density of 1 × 10^4^ cells/well in 96-well plates and incubated at 37 °C with 5% CO_2_ for 24 h. After aspirating the supernatant, 100 μL of medium containing the PLL, HDCA, or PLL-HDCA NPs at concentrations of 20 μg/mL, 40 μg/mL, and 80 μg/mL was added to each well. The cells were incubated for 48 h, followed by the addition of 100 μL of 10% CCK-8/DMEM (*v*/*v*) to each well and incubation for another 2 h. Absorbance was measured at 450 nm using a microplate reader (Biotek Synergy H1, Winooski, VT, USA). Experiments were conducted in quadruplicate, and the cell survival ratio was calculated as:

Cell survival ratio (%) = (A_s_ − A_b_)/(A_c_ − A_b_) × 100% (4)

where A_s_ is the sample group OD value, A_c_ is the control group OD value, A_b_ is the blank group OD value.

Hemocompatibility was assessed via hemolysis assays by measuring red blood cell (RBC) lysis in the presence of PLL-HDCA NPs. Blood samples were collected from healthy mice into tubes containing 3.8% sodium citrate anticoagulant at a 9:1 *v*/*v* ratio of blood to anticoagulant. RBCs were separated and diluted six-fold with saline before incubating with PLL-HDCA NPs for 2 h. Negative controls (distilled water) and positive controls (saline) were included. After centrifugation at 3000 rpm for 3 min, supernatants were collected, and absorbance was measured at 540 nm. The hemolysis ratio was calculated as:

Hemolysis ratio (%) = (A_s_ − A_n_)/(A_p_ − A_n_) × 100% (5)

where A_s_ is the sample group OD value, A_p_ is the positive control group OD value, A_n_ is the negative group OD value.

Skin irritation tests were conducted to evaluate the safety of PLL-HDCA NPs. Male Kunming mice were randomly assigned to four groups: Saline, PLL, HDCA, and PLL-HDCA NPs. Hair on the backs of the mice was shaved, and the skin was checked to ensure it was free from edema, erythema, or damage. For the single-skin irritation test, saline was applied to one side of the back as a control, while PLL, HDCA, or PLL-HDCA NPs were applied to the other side. After 24 h, the treated areas were washed, and skin condition was evaluated at 2, 24, and 48 h post-treatment. The multiple-skin irritation test extended treatment to 7 consecutive days, with skin condition monitored and scored using predefined criteria.

### 2.9. Statistical Analysis

Statistical analysis was performed using GraphPad Prism 8.2.0 software (GraphPad Software, San Diego, CA, USA). Mean and standard deviations were calculated for all experimental groups. One-way ANOVA with Tukey’s multiple comparisons test was used for normally distributed flow cytometry data. For in vivo studies, bleeding time and blood loss were analyzed using the Mann–Whitney two-tailed test and the Kruskal–Wallis test and post hoc comparison. Statistical significance was indicated as * *p* < 0.05, ** *p* < 0.01, *** *p* < 0.001, or **** *p* < 0.0001.

## 3. Results and Discussion

### 3.1. Preparation and Characterization of PLL-Cholic Acid Derivative Nanoparticles

Hydrophilic polymers and hydrophobic drugs are known to form amphiphilic supramolecular complexes through adequate interactions between the polymer and drug, leading to the formation of nanoparticles [19,20,21]. To investigate the morphological characteristics of PLL-cholic acid derivative complexes, nanocomplexes were synthesized via PLL-mediated self-assembly of CHE, CA, DCA, HDCA, GDCA, OBE, TDCA, TCA, TSCA, and UDCA, following the nanofabrication procedure.

Among the synthesized nanocomplexes, only PLL-HDCA, PLL-OBE, and PLL-CHE formed well-defined nanoparticles with a narrow size distribution, and no agglomeration was observed. In contrast, the other complexes appeared as clear solutions with minimal nanoparticle formation detectable in the characterization tests, suggesting that these complexes did not form regular nanoparticles and were excluded from further analysis. Additionally, PLL-DCA precipitated from the solution after standing overnight (Figure 2a). The particle sizes of PLL-HDCA, PLL-OBE, and PLL-CHE were approximately 200 nm, 300 nm, and 400 nm, respectively (Figure 2h). TEM images revealed that PLL-HDCA, PLL-OBE, and PLL-CHE nanoparticles were uniform, spherical granules (Figure 2g). A count rate of 1600–1800 kps was measured (Figure 2e), and the zeta potential ranged from 30 to 40 mV (Figure 2f). These results indicate that only HDCA, OBE, and CHE formed stable nanoparticles when coated with PLL.

### 3.2. Computer Simulation

To better understand the formation principles and processes of nanoparticles formed by cholic acid derivatives and PLL, we employed multiscale computer simulations. By integrating molecular simulations with experimental techniques, theoretical simulations and experimental results were able to support and complement each other. Adsorption energy values, calculated through molecular docking (Figure 2b), revealed that the adsorption energy of cholic acid derivatives to PLL was higher than their self-association energy, indicating a preference for binding to PLL. Notably, the PLL-DCA complex precipitated due to its lower adsorption energy, as shown in Figure 2a,b. These theoretical calculations corresponded well with the experimental outcomes.

The Blends module in Material Studio 8.0 software was used to evaluate intermolecular compatibility by calculating the Chi (Flory–Huggins interaction parameter, χ) value [22]. Lower Chi values indicate better compatibility between two molecules. For instance, the calculated Chi values were as follows: χ _(LL-HDCA)_ = 5.228, χ _(LL-water)_ = 7.07, and χ _(water-HDCA)_ = 7.878, suggesting that HDCA exhibits higher compatibility with L-lysine (LL) than with water. As summarized in Table 1, the Chi value for all cholic acid derivatives indicates a preference for binding to PLL over water.

Through hydrophilic and hydrophobic surface mapping of the ten complexes, we observed evident amphiphilic properties, as shown in Figure 2c. The green regions represent hydrophobic areas, while the purple regions denote hydrophilic areas. Our findings revealed that cholic acid derivatives combined with PLL to form amphiphilic complexes, where the cholic acid derivatives constituted the hydrophobic region and PLL formed the hydrophilic region. According to the amphiphilic map generated using ImageJ software, CHE, OBE, and HDCA exhibited a significantly higher proportion of hydrophobic areas (green), while the other derivatives displayed a greater hydrophilic ratio (purple), as shown in Figure 2d. This amphiphilic property enhances the water solubility of these complexes, allowing them to dissolve in water. These results are consistent with previous observations in Figure 2a.

The energy profile from molecular dynamics (MD) simulations demonstrated notable stability throughout the simulation, as shown in Figure S2. The logP value, an indicator of hydrophobicity, reflects the logarithmic value of the partition coefficient of a substance between octanol and water. Higher logP values indicate greater hydrophobicity. The logP values of cholic acid derivatives showed that OBE, HDCA, DCA, CHE, and UDCA exhibited relatively higher hydrophobicity, aligning closely with the hydrophobicity proportions observed in MD simulation results (Figure S1).

In theory, molecules combine through various intermolecular forces, including π-π stacking, hydrogen bonding, ionization, and van der Waals forces. However, it is challenging to predict whether these interactions result in stable nanoparticles. By employing dissipative particle dynamics (DPD) simulations, we simplified molecular interactions into particle-level forces, facilitating the prediction of stable nanoparticle formation. Based on previous simulation results, CHE, OBE, and HDCA demonstrated the ability to self-assemble, making them ideal candidates for DPD simulations with PLL.

Initially, PLL and cholic acid derivatives were randomly distributed within the simulation system. Over time, the molecules self-assembled into small nanoparticles that grew into stable spherical core–shell structures. Due to PLL’s hydrophilic nature and the hydrophobicity of cholic acid derivatives, PLL formed the outer shell, while the cholic acid derivatives formed the core. Figure 2i illustrates the successful self-assembly of CHE, OBE, and HDCA with PLL into stable nanoaggregates, consistent with previous findings (Figure 2a).

#### 3.2.1. Confirmation of Intermolecular Interactions

**Figure 3:**
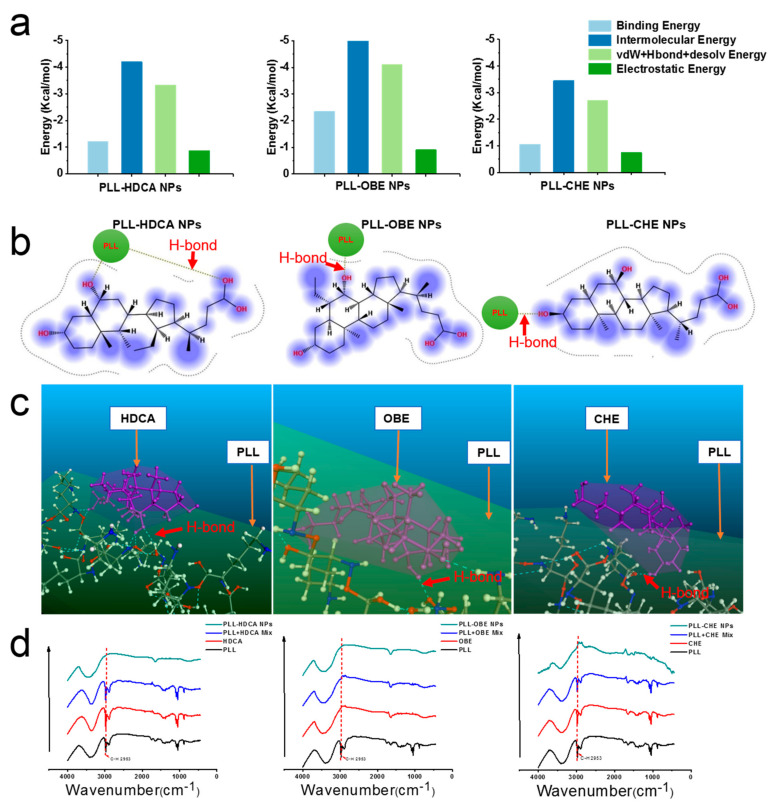
Molecular modeling and experimental characterization of interactions between PLL and HDCA, OBE, CHE. (**a**) Various binding energies calculated by Autodock 4.2. The binding interaction mainly depends on van der Waals force and electrostatic interaction. (**b**) 2D images displaying the lowest energy conformation and the interaction of PLL and HDCA, OBE, CHE, respectively. (**c**) The lowest energy 3D conformation of PLL/HDCA, OBE, CHE complexes. The Blue dotted line (red arrows) indicates H-bonding. (**d**) Characterization of PLL/HDCA, OBE, CHE forces by FT-IR spectroscopy.

To confirm the ability of hydrophilic PLL and hydrophobic drugs (CHE, OBE, and HDCA) to form amphiphilic nanoparticles, molecular simulations, including docking, MD, and DPD, were employed. Using Materials Studio, Autodock, and MOE software, we investigated the intermolecular forces involved in nanoparticle formation, as shown in Figure 3a–c. Hydrogen bonding between the alkyl group of PLL and the hydroxyl group of HDCA, OBE, and CHE confirmed the presence of strong intermolecular interactions.

The interaction between PLL and cholic acid derivatives was further validated using infrared spectroscopy (Figure 3d). The spectrum of PLL exhibited a broad peak between ~3600 cm^−1^ and ~3058 cm^−1^, attributed to overlapping peaks of amine (-NH_2_, -NH-) and hydroxyl (-OH) stretching vibrations [23]. Additionally, PLL’s C–H bonds showed stretching vibrations at ~2953 cm^−1^ [24,25,26]. In the PLL-HDCA, PLL-CHE, and PLL-OBE nanoparticles, the C–H absorption peak (~2953 cm^−1^) was significantly reduced, indicating hydrogen bonding and hydrophobic interactions between the cholic acid derivatives and PLL [27]. These findings, supported by molecular simulations and experimental data, confirmed robust intermolecular interactions responsible for forming well-structured nanoparticles.

#### 3.2.2. Selection of PLL-HDCA Nanoparticles for Further Investigation

Previous studies [28] have demonstrated that HDCA possesses antibacterial properties, aligning with our objective of achieving hemostasis while preventing bacterial infections. Study [29] indicates that nanoparticles with a size of 140–220 nm exhibit enhanced platelet affinity, while larger particles (>220 nm) show reduced binding capacity. Furthermore, nanoparticles with greater surface charge promote platelet aggregation more effectively. Given that the particle size of PLL-HDCA NPs is approximately 200 nm, while PLL-CHE NPs and PLL-OBE NPs are around 300–400 nm, PLL-HDCA NPs were selected for further investigation due to their favorable characteristics.

### 3.3. Characterization of PLL-HDCA NPs

**Figure 4:**
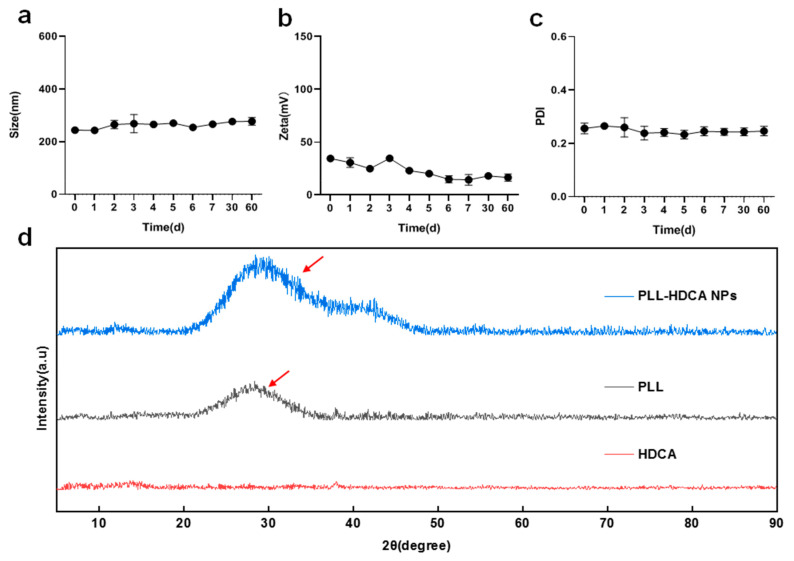
The characterization of PLL-HDCA NPs. (**a**–**c**) The stability of PLL-HDCA NPs was examined by evaluating the particle size (**a**), zeta potential (**b**), and PDI (**c**). (**d**) XRD patterns of HDCA, PLL, and PLL-HDCA NPs. The red arrows indicate the peak position. Data are mean ± SD (*n* = 3).

The stability of PLL-HDCA NPs was evaluated by monitoring their particle size, zeta potential and PDI over 60 days. The results showed minimal changes in these parameters, indicating that PLL-HDCA NPs exhibit good stability (Figure 4a–c). XRD analysis was performed to verify the phase composition of PLL-HDCA NPs. The XRD patterns (Figure 4d) showed that PLL-HDCA NPs exhibited similar patterns to PLL. While the peak positions and shapes were consistent, the intensity of the peaks increased, confirming the presence of both PLL and HDCA in the nanoparticles. After adding HDCA, the peak intensity of PLL-HDCA was higher than that of PLL, indicating that the addition of HDCA can effectively enhance the crystallinity of PLL [30].

Drug loading and encapsulation efficiency were determined using high-performance liquid chromatography (HPLC). The results revealed a drug loading capacity of 65% for HDCA and an encapsulation efficiency of 91%.

### 3.4. In Vitro Hemostatic Performance of PLL-HDCA NPs

**Figure 5:**
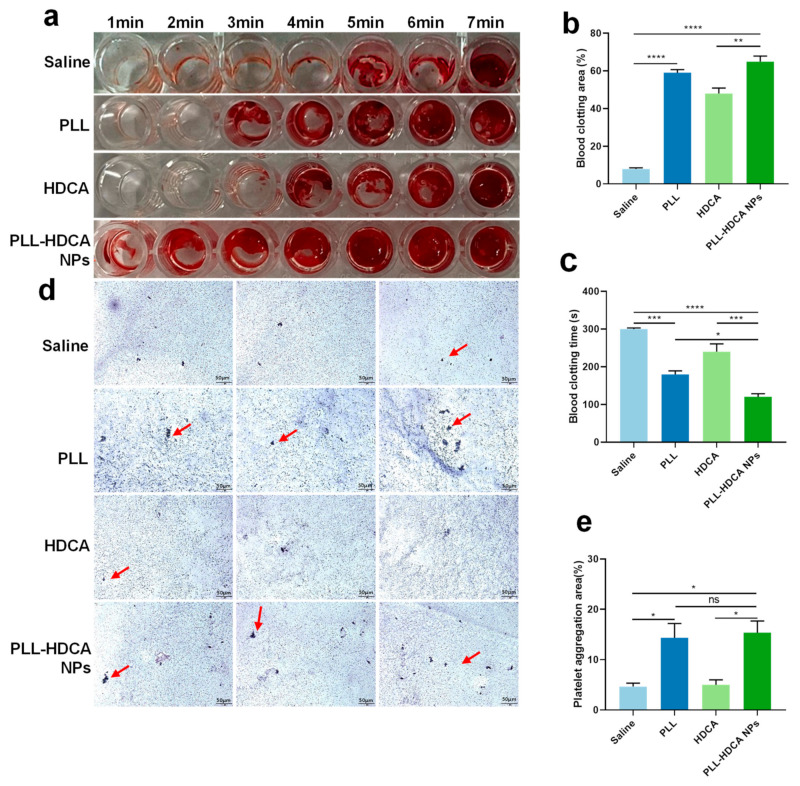
Hemostatic properties of PLL-HDCA NPs in vitro. (**a**) The representative image of coagulation assay. (**b**) Proportion of blood clotting area of various samples. (**c**) Statistical analysis of blood clotting time of various samples. (**d**) Fluorescence microscope images of platelets after being treated with various specimens. The red arrows indicate aggregated platelets. (**e**) Statistical analysis of platelet aggregation area. Data are mean ± SD (*n* = 3) of independent experiments; * *p* < 0.05, ** *p* < 0.01, *** *p* < 0.001, and **** *p* < 0.0001, ns = not significantly different.

The screening study demonstrated that PLL-HDCA NPs possess favorable properties as a nano-drug. To evaluate their potential to promote blood coagulation, whole blood was co-incubated with the hemostatic agents. The in vitro hemostatic ability of PLL-HDCA NPs was assessed by measuring whole blood coagulation time and clotting area. Figure 5a–c showed that the coagulation time of PLL-HDCA NPs was significantly shorter than that of the control groups (normal saline, PLL, and HDCA at concentrations corresponding to PLL at 1 mg/mL). Specifically, coagulation initiated at 1 min for PLL-HDCA NPs, 3 min for PLL, 4 min for HDCA, and 5 min for saline. Notably, the clotting area of PLL-HDCA NPs was the largest among all groups (Figure 5b).

Although PLL alone can promote platelet aggregation, the results suggest that assembled PLL-HDCA NPs exhibit significantly hemostatic effects. These findings indicate that the formation of PLL-HDCA NPs substantially accelerates the coagulation process, resulting in rapid hemostasis.

After physical injury, platelet aggregation plays a critical role in hemostasis, as platelets are activated by coagulation factors. The adhesion and activation of PLL on platelets have been known for nearly half a century and are frequently used in platelet activation models [31,32]. To explore the mechanism underlying nanoparticle-mediated hemostasis, the platelet-activating ability of PLL-HDCA NPs was examined using electron microscopy. As shown in Figure 5d,e, PLL-HDCA NPs induced more platelet aggregation in platelet-rich plasma (PRP) compared to HDCA and saline.

The study found no significant difference between PLL-HDCA NPs and PLL in their ability to induce platelet aggregation, while HDCA alone exhibited minimal effects. These results indicate that PLL-HDCA NPs effectively inherit the platelet activation capability of PLL, thereby enhancing platelet aggregation. In conclusion, PLL-HDCA NPs demonstrated effective in vitro hemostatic performance by promoting platelet aggregation, suggesting their potential for rapid blood coagulation.

### 3.5. In Vivo Hemostatic Effect of PLL-HDCA NPs

**Figure 6:**
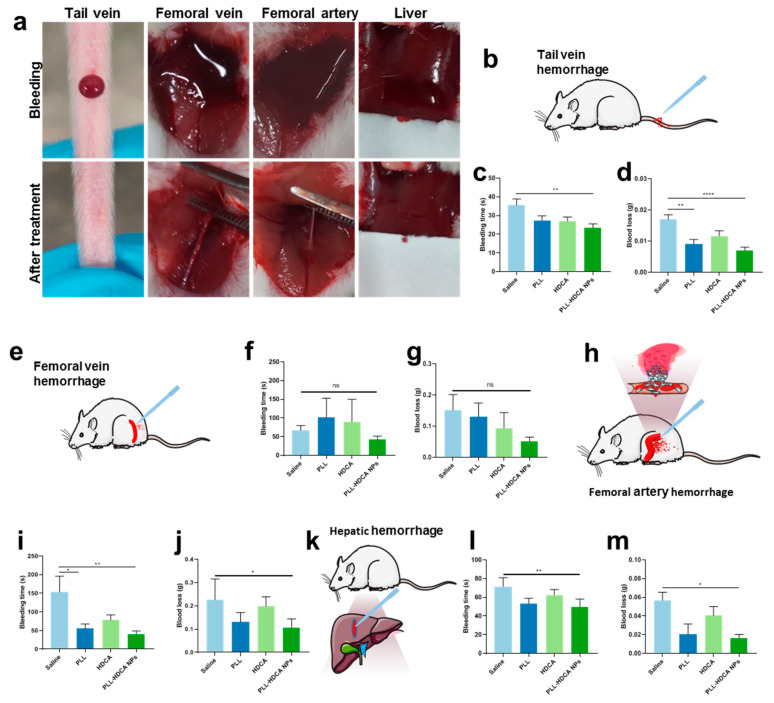
Hemostatic effects of topical administrations of PLL-HDCA NPs in different bleeding models. (**a**) Digital photos of hemorrhage at various stages before and after treatment with PLL-HDCA NPs. (**b**–**d**) Hemostatic action in tail vein hemorrhage model. (**b**) Schematic illustration, (**c**) bleeding time, and (**d**) blood loss in vein hemorrhage model. (**e**–**g**) Hemostatic action in femoral vein hemorrhage model. (**e**) Schematic illustration, (**f**) bleeding time, and (**g**) blood loss in femoral vein hemorrhage model. (**h**–**j**) Hemostatic action in femoral artery hemorrhage model. (**h**) Schematic illustration, (**i**) bleeding time, and (**j**) blood loss in femoral artery hemorrhage model. (**k**–**m**) Hemostatic action in hepatic hemorrhage model. (**k**) Schematic illustration, (**l**) bleeding time, and (**m**) blood loss in hepatic hemorrhage model. Data are mean ± SD (*n* = 5) of independent experiments; * *p* < 0.05, ** *p* < 0.01, and **** *p* < 0.0001, ns = not significantly different.

Based on the previous studies, we investigated the in vivo hemostatic effects of PLL-HDCA NPs under various bleeding conditions. The mouse tail vein hemorrhage model, widely regarded as an ideal trauma model, sensitively reflects the in vivo efficacy of coagulation drugs [33]. The femoral artery and vein hemorrhage models are utilized to evaluate the hemostatic efficacy in cases of massive and compressible bleeding models, respectively [15]. Meanwhile, the liver hemorrhage model serves as a representative non-compressible model for severe internal bleeding and intraoperative hemorrhage [34]. As illustrated in Figure 6, mice treated with PLL-HDCA NPs exhibited significantly shorter bleeding times and reduced blood loss compared to other groups across all four hemorrhage models, at a concentration corresponding to PLL (1 mg/mL). These results indicate that PLL-HDCA NPs effectively reduced blood loss and shortened bleeding times across multiple hemorrhage types via topical administration, demonstrating robust in vivo hemostatic efficacy.

The hemostatic effect of PLL primarily relies on its amino groups, which are positively charged under physiological conditions, endowing PLL with strong cationic properties [35]. These positively charged amino groups interact with negatively charged components such as cell membranes and proteins, including fibrinogen, facilitating platelet activation and aggregation. When formulated into PLL-HDCA NPs, the positive charges of PLL are fully exposed on the nanoparticle surface, significantly enhancing its hemostatic performance. This structural feature enables the nanoparticles to effectively interact with the wound environment, promoting rapid coagulation and reducing bleeding time. Thus, the nanoscale formulation of PLL not only retains its inherent hemostatic properties but also optimizes their efficacy by leveraging the exposed surface charge of the nanoparticles.

### 3.6. Antibacterial Activities of PLL-HDCA NPs

In traumatic injuries, hemorrhage and bacterial infection frequently coexist, as wounds are highly susceptible to bacterial contamination [36]. Therefore, an effective hemostatic agent should also possess antibacterial properties to protect wound tissue from external infections. Since PLL is a natural antibacterial substance, the antibacterial properties of PLL-HDCA NPs were further explored.

The antibacterial efficacy of PLL-HDCA NPs were assessed by *S. aureus* and *E. coli*, which commonly occur during the early and late stages of infection, respectively. Turbidity tests of bacterial cultures revealed that the minimum inhibitory concentration (MIC) of PLL-HDCA NPs against *E. coli* and *S. aureus* was 0.375 mg/mL and 0.1875 mg/mL, respectively (Figure S5).

**Figure 7:**
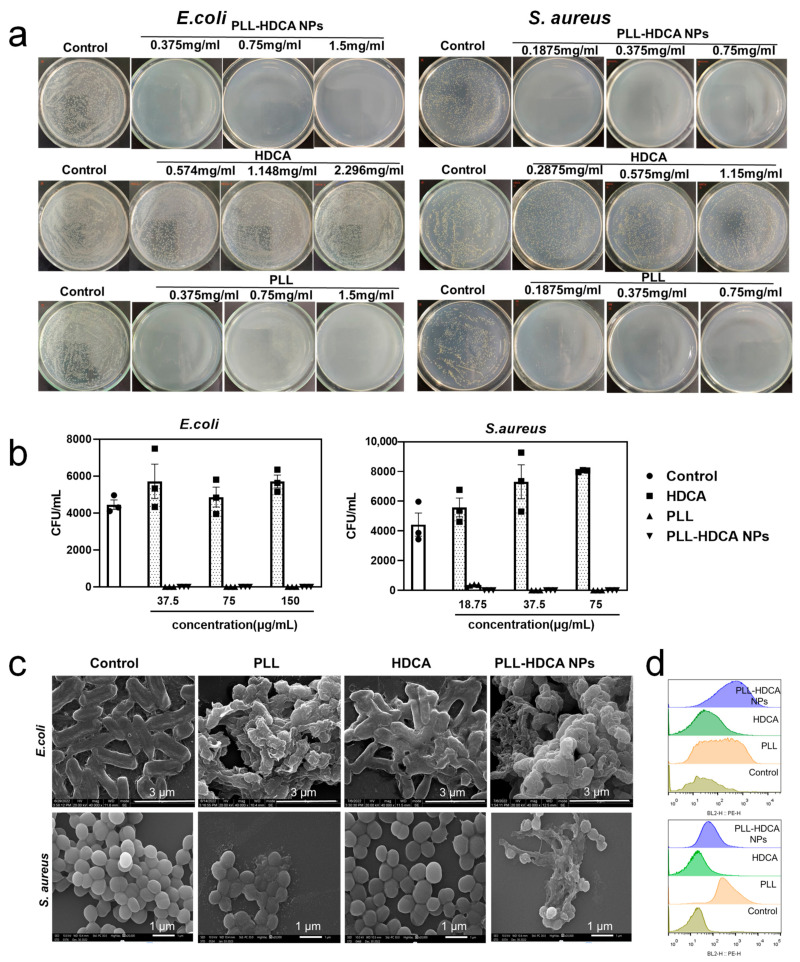
Antibacterial activity of PLL-HDCA NPs. (**a**) Plate counting images of *E. coli* and *S. aureus* after treatment with HDCA, PLL, PLL-HDCA NPs, using PBS treatment as control. (**b**) Statistical analysis of *E. coli* and *S. aureus* viable count after treatment with HDCA, PLL, PLL-HDCA NPs, using PBS treatment as control. (**c**) Morphology of *E. coli* and *S. aureus* after co-culturing with samples at a concentration corresponding to the PLL (0.375 mg/mL)/(0.1875 mg/mL), respectively. (**d**) The integrity of *E. coli* and *S. aureus* cell membrane was determined by flow cytometry after co-culturing with samples at a concentration corresponding to the PLL (0.375 mg/mL)/(0.1875 mg/mL), respectively. Data are mean ± SD (*n* = 3).

As shown in Figure 7a,b, the number of viable bacteria in the control and HDCA-treated groups was significantly higher than in the PLL and PLL-HDCA NPs groups, indicating that PLL-HDCA NPs effectively eliminated *S. aureus* and *E. coli*. These findings confirm that PLL-HDCA NPs inherit the strong antibacterial properties of PLL against both *S. aureus* and *E. coli*.

Morphological changes in *S. aureus* and *E. coli* were observed on the surfaces of various samples. Compared to the control and HDCA groups, bacteria exposed to PLL-HDCA NPs exhibited damaged cellular morphology (Figure 7c). Propidium iodide (PI), a membrane-impermeant fluorophore, was used to tag bacterial cells with compromised membranes. After staining with PI, changes in fluorescence intensity were measured via flow cytometry to assess the extent of membrane damage. As shown in Figure 7d, the cell membrane damage rate for *E. coli* in the PLL-HDCA NPs, PLL, HDCA, and control groups was 36.5%, 18.2%, 2.26%, and 1.99%, respectively. For *S. aureus*, the corresponding rates were 39.8%, 19.1%, 8.94%, and 7.13%.

Combining the results of SEM observations and flow cytometry, we speculate that the antibacterial activity of PLL-HDCA NPs is related to their positive charge. The cationic charge of PLL facilitates the adsorption of negatively charged bacteria [37,38], disrupting energy metabolism, respiration, and electron transport systems, ultimately leading to bacterial death [23]. Furthermore, PLL disrupts bacterial cell wall and membrane components, significantly increasing cell wall permeability and causing leakage of protoplasm, which results in cell death [39]. Additionally, PLL induces the accumulation of intracellular reactive oxygen species (ROS) and DNA fragmentation, further contributing to bacterial cell death [40,41,42].

Although HDCA alone did not exhibit significant antibacterial effects as previously reported [15], this may be attributed to its reliance on synergistic interactions with other components for antibacterial activity. Fortunately, the combined application of PLL and HDCA in PLL-HDCA NPs demonstrated strong antibacterial properties.

### 3.7. In Vivo Wound-Healing Assessment for PLL-HDCA NPs

During the wound healing process, bacterial infections can delay recovery. Nutrient and oxygen supply through new blood vessels is critical for successful wound repair and overall health [43,44]. Therefore, essential factors for effective wound healing include a sterile environment free of infections and a conducive microenvironment that supports tissue growth and vascularization.

**Figure 8:**
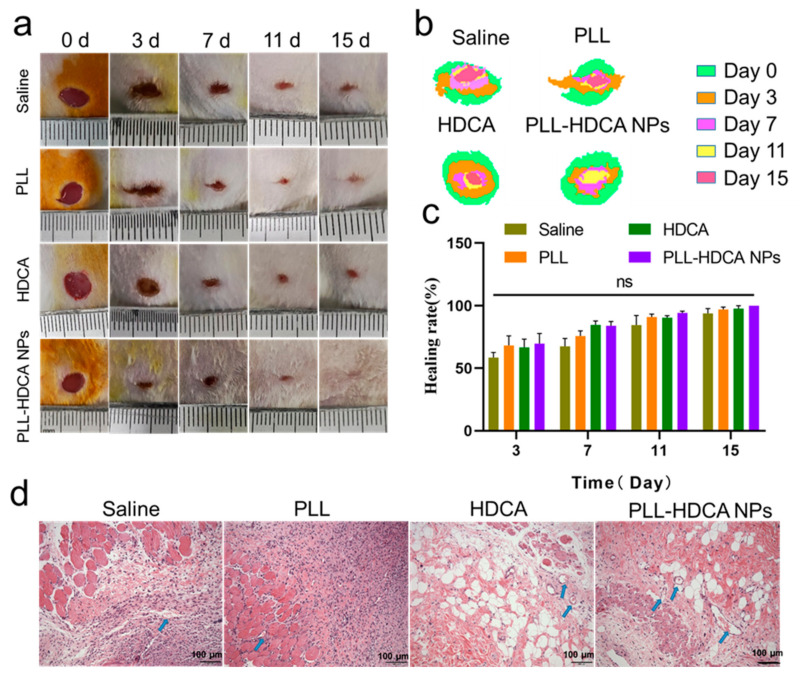
Wound healing assay to evaluate wound closure ability of PLL-HDCA NPs at different time points. (**a**) Morphology of wound healing at different time points. (**b**) Schematic diagram that mimicking wound healing process. (**c**). Wound healing rate of the samples at the set time point. (**d**) H&E-stained pictures of wound skin tissue on days 7 after treatment (scale bar = 100 μm). Blue arrows represent newly generated blood vessels. Data are mean ± SD (*n* = 3) of independent experiments; ns = not significantly different.

As shown in Figure 8a, experimental results demonstrated that PLL-HDCA NPs significantly accelerated wound closure compared to the Saline, PLL, and HDCA groups. In Figure 8b, a superimposed schematic diagram of wound healing over time (days 0, 3, 7, 11, and 15) revealed that although all groups exhibited some degree of wound area reduction; the PLL-HDCA NPs group showed the most pronounced progress, achieving rapid wound repair.

Figure 8c illustrates that on days 3, 7, 11, and 15 of the experiment, the wound healing rate in the PLL-HDCA NPs group was higher compared to the Saline, PLL, and HDCA groups. Although the differences between PLL-HDCA NPs and the other groups were not statistically significant, the continued acceleration of wound area reduction suggests the potential of PLL-HDCA NPs in promoting wound healing.

To further evaluate wound remodeling, wound tissues from each group were collected on day 7 and analyzed using H&E staining. As shown in Figure 8d, the H&E-stained sections revealed that PLL-HDCA NPs significantly enhanced fibroblast proliferation and the formation of numerous newly generated blood vessels (indicated by blue arrows) compared to the Saline, PLL, and HDCA groups. These findings suggest that PLL-HDCA NPs exhibit superior neovascularization capacity. The restorative effect of PLL-HDCA NPs may be attributed to their antibacterial properties, which create a sterile environment, reduce bacterial invasion and inflammation, and ultimately promote wound healing [45].

### 3.8. Evaluation of Biocompatibility of PLL-HDCA NPs

**Figure 9:**
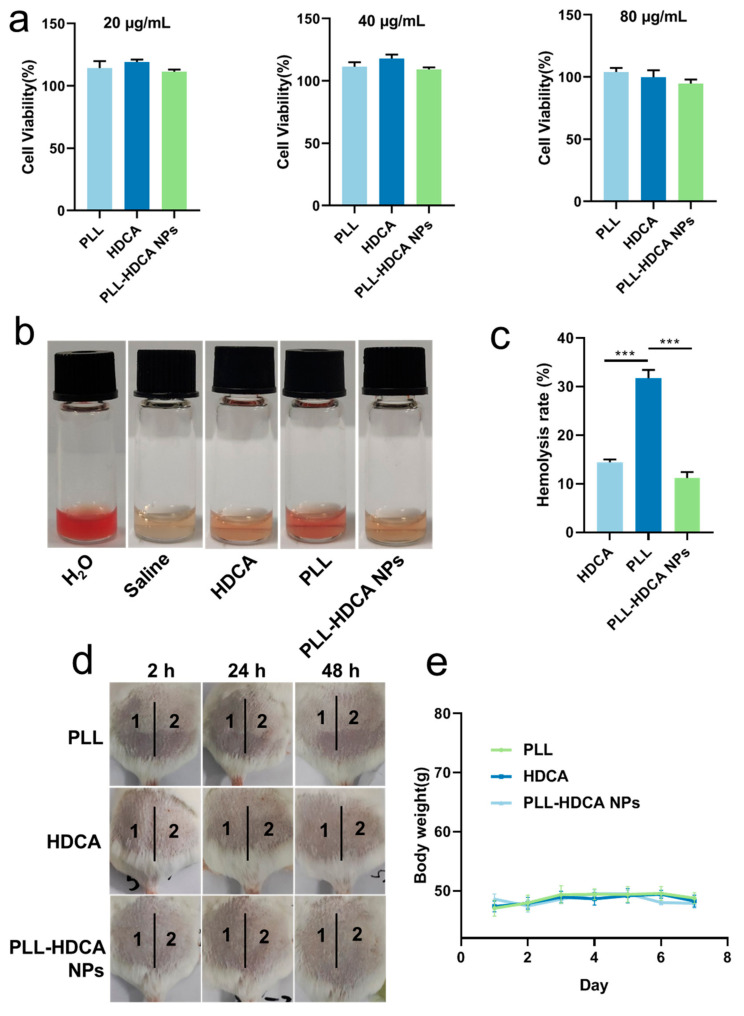
Biocompatibility test of PLL-HDCA NPs. (**a**) Cytotoxicity assay of PLL-HDCA NPs. (**b**) Hemolysis assay of PLL-HDCA NPs. (**c**) Hemolysis rate statistic of PLL-HDCA NPs. (**d**) Changes in skin appearance of mice after skin irritation test: the normal saline (zone 1), PLL/HDCA/PLL-HDCA NPs (zone 2). (**e**) Changes in body weight of mice after skin irritation test. Data are mean ± SD (*n* = 3) of independent experiments; *** *p* < 0.001.

Fibroblasts play a crucial role in wound healing and reconstruction, making them a reliable model for evaluating cytocompatibility. Human skin fibroblast (HSF) cells were employed to assess the cytotoxicity of PLL-HDCA NPs. As shown in Figure 9a, the viability of HSF cells remained high (95–120%) when treated with 20–80 μg/mL of PLL-HDCA NPs, indicating that these nanoparticles exhibit no toxicity toward human fibroblasts.

Since hemostatic agents directly contact blood, it is critical that they do not induce hemolysis or damage red blood cells (RBCs) [26]. The hemolytic activity of PLL-HDCA NPs was evaluated by treating RBCs with PLL-HDCA NPs, HDCA, and PLL for 2 h. As shown in Figure 9b, PLL-HDCA NPs exhibited a significantly lower hemolysis rate than PLL alone at equivalent dosages (*p* < 0.001). This reduction is likely due to the carboxyl groups of HDCA shielding the amino groups of PLL, thereby enhancing safety. While the observed hemolysis rate of 11% may limit intravenous use, PLL-HDCA NPs remain viable for local applications as sprays, dressings, or liquid- or gel-based formulations for hemostasis and antibacterial purposes.

Additionally, PLL-HDCA NPs hold potential for further development through optimizing the ratio, concentration, biodistribution, and release mechanisms to better balance coagulation and hemolysis within the body. Although HDCA alone did not significantly improve the hemostatic or antibacterial properties of the nanoparticles, it effectively reduced the hemolytic toxicity of PLL, which tends to adsorb onto RBC surfaces due to its strong cationic nature. This combination substantially reduced the hemolytic effects of PLL.

In the single administration skin irritation test, the test areas on the backs of mice were scored and evaluated according to Tables S2 and S3. The results are summarized in Table 2. For all tested groups (Saline, PLL, HDCA, and PLL-HDCA NPs), no erythema or edema was observed at 2, 24, or 48 h post-administration, with an average irritation score of zero. These results indicate that none of the tested materials caused skin irritation.

In the multiple administration skin irritation test, mice were treated daily for seven consecutive days, and their backs were evaluated according to Tables S2 and S3. Results are presented in Table 3 and Figure 9d,e. For Saline, PLL, and PLL-HDCA NPs, the irritation scores remained zero throughout the experiment, with no erythema or edema observed. In the HDCA group, a minor average score of 0.33 was recorded at 2 and 24 h post-treatment removal, indicating negligible irritation (scores below 0.5 are classified as non-irritating). Additionally, no significant changes in body weight were observed across all groups (Saline, PLL, HDCA, and PLL-HDCA NPs), as shown in Figure 9e. These results confirm the high safety profile of PLL-HDCA NPs, highlighting their potential for therapeutic applications and biomedical development.

Compared to reported UDCA/bPEI25 nanoparticles [15], the PLL-HDCA NPs demonstrate distinct innovations and advantages. First, unlike UDCA/bPEI25 nanoparticles, which are primarily administered via injection, our research focuses on local administration. This approach significantly improves the safety profile by avoiding the adverse effects commonly associated with injectable polyethyleneimine (PEI)-based systems, such as toxicity, thrombotic risks, and severe side effects at higher doses. Second, PLL not only provides hemostatic properties but also exhibits significant antibacterial activity, unlike PEI. The inherent safety concerns of PEI limit its clinical applications, whereas PLL-HDCA NPs overcome these limitations, enhancing their feasibility for clinical translation. Together, these findings position PLL-HDCA NPs as a safer and more versatile alternative for localized drug delivery and wound care.

## 4. Conclusions

In summary, multifunctional drugs with hemostatic, antibacterial, and wound-healing properties are essential for improving treatment efficiency in resource-limited environments and addressing complex injuries at trauma first-aid sites. This study confirmed that PLL can self-assemble with cholic acid derivatives (HDCA, CHE, and OBE) into well-structured nanoparticles through multiple intermolecular interactions. Notably, PLL-HDCA nanoparticles (NPs) demonstrated rapid blood coagulation within 1 min, inhibited the growth of *E. coli* and *S. aureus*, and exhibited comprehensive hemostatic, antibacterial, and wound-healing effects.

Compared to similar nanoparticle systems [46], PLL-HDCA NPs offer distinct advantages. They are specifically designed for localized application, reducing systemic toxicity and improving safety profiles while maintaining multifunctional properties. Unlike PEI-based systems, which lack inherent antibacterial effects, PLL-HDCA NPs effectively integrate both hemostatic and antibacterial functionalities, addressing the critical need for infection control and wound repair simultaneously.

The reduced hemolytic activity of PLL-HDCA NPs compared to PLL alone highlights the importance of HDCA in modulating toxicity while preserving hemostatic efficacy. Furthermore, their adaptability to various formulations, such as sprays, dressings, liquids, or gels, makes PLL-HDCA NPs a versatile candidate for diverse wound care scenarios, particularly in clinical and emergency settings.

These findings not only validate the innovative use of PLL-HDCA NPs for addressing the dual challenges of hemostasis and infection control but also provide a foundation for future studies to optimize nanoparticle formulations and extend their application to broader clinical settings. By integrating these multifunctional properties into a single nanoparticle system, PLL-HDCA NPs represent a significant advancement in the development of safe and effective therapeutic agents for managing infected wounds.

## Figures and Tables

**Table 1:** The Flory-Huggins interaction parameters calculated by blends (Unit: Kcal/mol).

Energies	Chi (298 K)	Emix (298 K)	Ebb Avg (298 K)	Ebs Avg (298 K)	Ess Avg (298 K)
LL-CHE	4.697	2.781	−4.174	−4.130	−5.255
LL-CA	5.191	3.074	−4.174	−4.214	−5.671
LL-DCA	−0.730	−0.432	−4.174	−4.150	−4.332
LL-GDCA	−10.251	−6.071	−4.174	−6.768	−7.698
LL-OBE	−11.245	−6.659	−4.174	−6.522	−7.081
LL-TDCA	−2.142	−1.268	−4.174	−6.025	−8.127
LL-TCA	6.140	3.636	−4.174	−6.922	−11.726
LL-TSCA	5.884	3.484	−4.174	−4.126	−5.820
LL-UDCA	−2.564	−1.519	−4.174	−5.201	−6.004
LL-HDCA	5.228	3.096	−4.174	−3.577	−4.345
LL-H_2_O	7.072	4.188	−4.182	−4.182	−10.637
H_2_O-H_2_O	0.000	0.000	−2.311	−2.311	−2.311
H_2_O-CHE	11.748	6.957	−2.311	−1.985	−5.255
H_2_O-CA	12.203	7.227	−2.311	−2.055	−5.671
H_2_O-DCA	5.228	3.096	−2.311	−2.121	−4.332
H_2_O-GDCA	18.024	10.673	−2.311	−2.355	−7.698
H_2_O-OBE	8.837	5.233	−2.311	−2.856	−7.081
H_2_O-TDCA	10.190	6.034	−2.311	−3.144	−8.127
H_2_O-TCA	20.889	12.370	−2.311	−3.626	−11.726
H_2_O-TSCA	19.278	11.416	−2.311	−1.517	−5.820
H_2_O-UDCA	8.660	5.129	−2.311	−2.491	−6.004
H_2_O-HDCA	7.878	4.665	−2.311	−1.913	−4.345

**Table 2:** Single administration skin irritation experiments results.

Treatment	Integral Mean			Stimulus Intensity		
	2 h	24 h	48 h	2 h	24 h	48 h
Saline	0	0	0	-	-	-
PLL	0	0	0	-	-	-
HDCA PLL-HDCA NPs	0 0	0 0	0 0	- -	- -	- -

“-” represented no skin irritation.

**Table 3:** Multiple administration skin irritation experiments results.

Treatment	Integral Mean			Stimulus Intensity		
	2 h	24 h	48 h	2 h	24 h	48 h
Saline	0	0	0	-	-	-
PLL	0	0	0	-	-	-
HDCA PLL-HDCA NPs	0.33 0	0.33 0	0 0	- -	- -	- -

## Data Availability

All data needed to evaluate the conclusions in the paper are present in the paper and/or the Supplementary Materials.

## Supplementary Materials

The following supporting information can be downloaded at: https://www.mdpi.com/article/10.3390/pharmaceutics17010007/s1, Figure S1: LogP values of cholic acid derivatives; Figure S2: The dynamic energies change in MD simulation of PLL complex; Figure S3: The beads setting profile in DPD simulation; Figure S4: The dynamic energies change in DPD simulation of PLL complex; Figure S5: MIC of PLL-HDCA NPs and PLL to *E. coli* and *S. aureus*. Table S1: The interaction parameters used in DPD simulations (unit: kT). Table S2: skin irritation response scoring criteria. Table S3. Evaluation criteria for skin irritation intensity.
