# Supplementary material for: Computer-Aided Construction and Evaluation of Poly-L-Lysine/Hyodeoxycholic Acid Nanoparticles for Hemorrhage and Infection Therapy
Source: Pharmaceutics. 2024 Dec 24;17(1):7. doi: 10.3390/pharmaceutics17010007 (PMC11768166; doi:10.3390/pharmaceutics17010007)
Supplement: Supplementary file 1 [file pharmaceutics-17-00007-s001.zip › pharmaceutics-3334816-supplementary.pdf]

Supplemental Materials

Computer-Aided Construction and Evaluation of Poly-L-Lysine/Hyodeoxycholic Acid Nanoparticles for Hemorrhage and Infection Therapy

Table S1. The interaction parameters used in DPD simulations (unit: kT).

	Chi	a_{ij}
LL-OBE	1. 549	83. 033
LL-CHE	2. 745	86. 923
LL-HDCA	2. 225	85. 232
H2O-OBE	14. 633	125. 558
H2O-CHE	14. 525	125. 207
H2O-HDCA	8. 467	105. 517
H2O-H2O	0	78
LL-H2O	2. 1051	84. 842

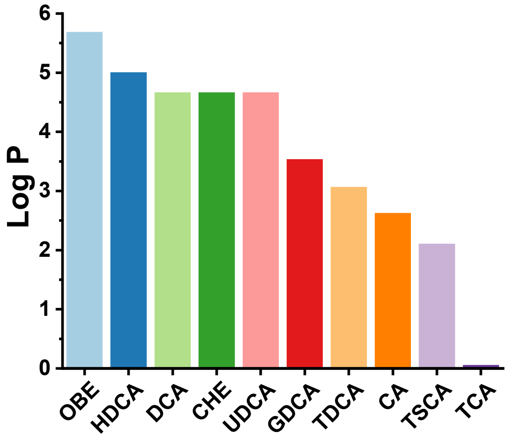

Figure S1. LogP values of cholic acid derivatives.

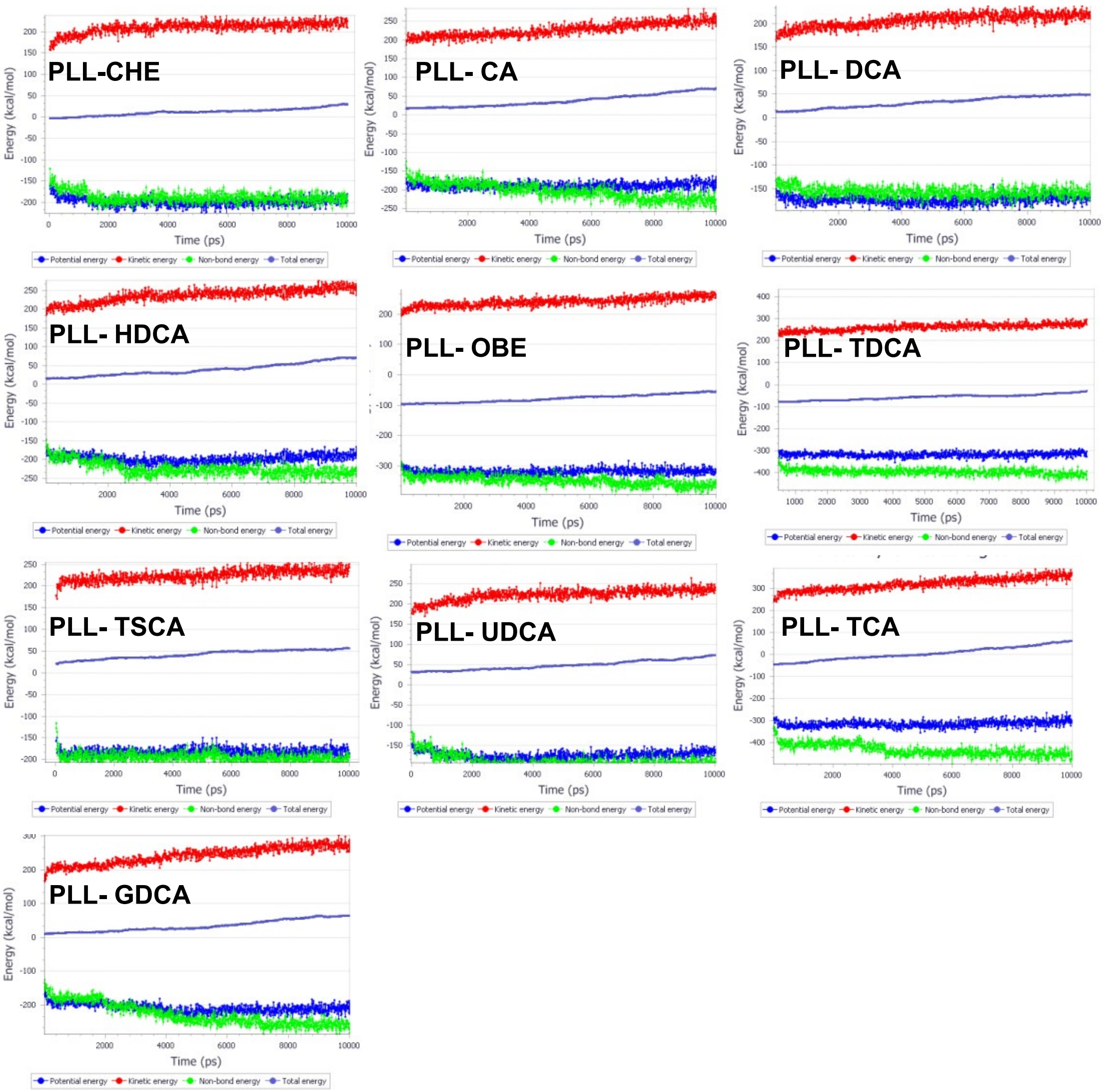

Figure S2. The dynamic energies change in MD simulation of PLL complex.

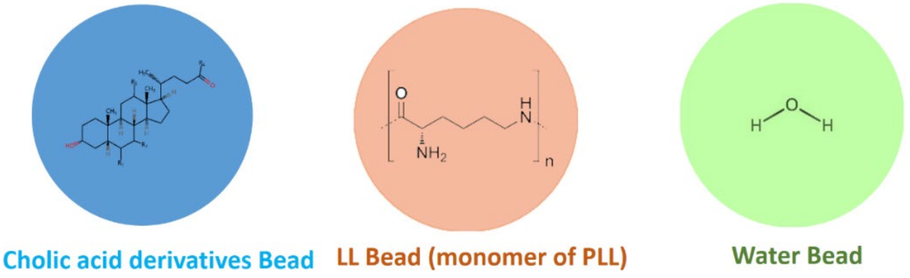

Figure S3. The beads setting profile in DPD simulation.

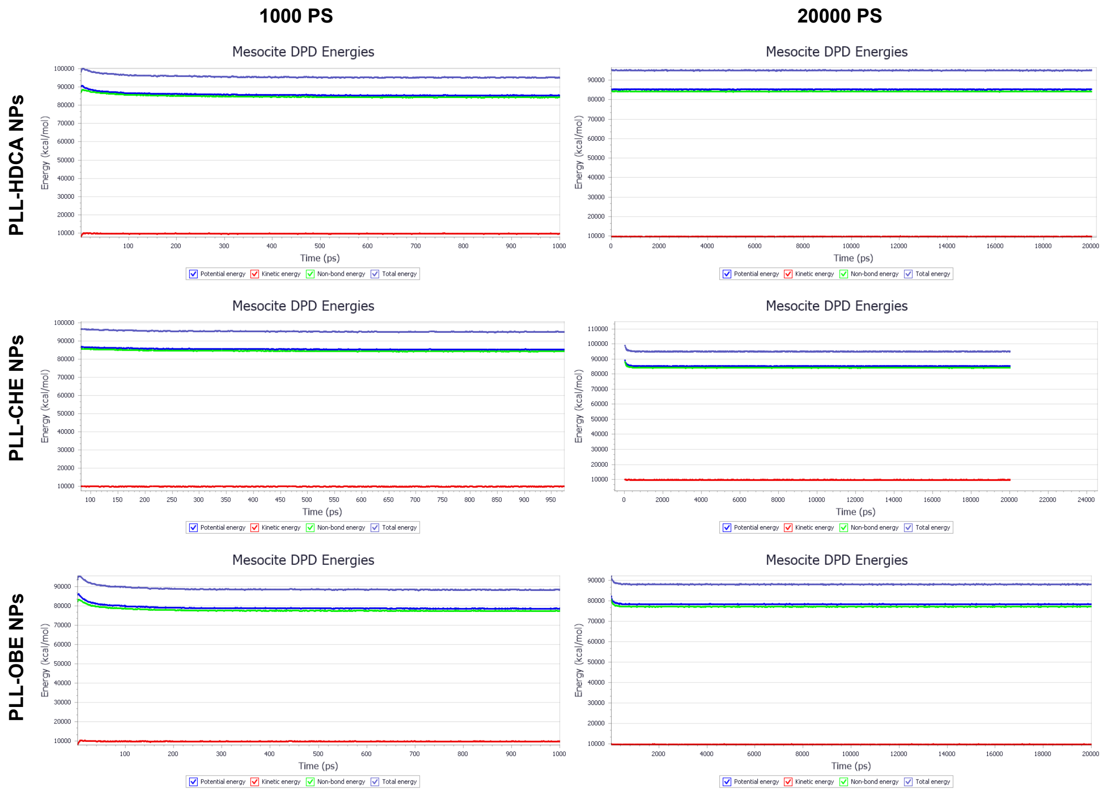

Figure S4. The dynamic energies change in DPD simulation of PLL complex.

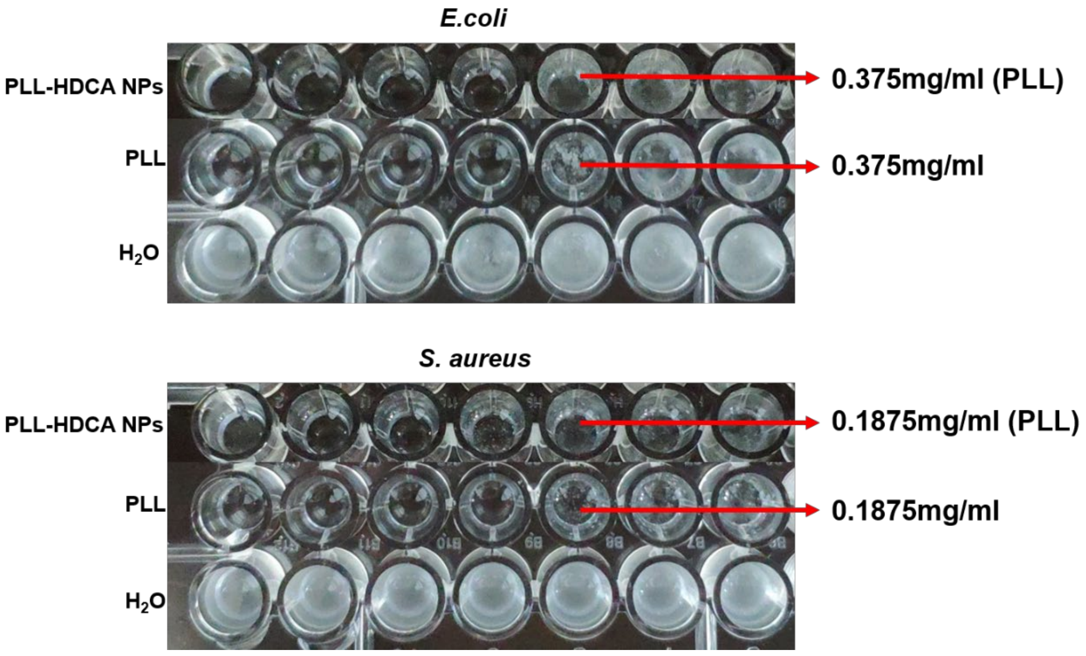

Figure S5. MIC of PLL-HDCA NPs and PLL to *E. coli* and *S. aureus*.

Table S 2. skin irritation response scoring criteria.

	Present	Score
Erythema		
	None present	0
	Mild erythema	1
	Moderate erythema	2
	Severe erythema	3
	Purplish red erythema with focal anthemia	4
Edema		
	None present	0
	Mild edema	1
	Moderate edema	2
	Severe edema	3
	Severe edema (edema swelling over 1mm)	4
	Total scores	8

Table S 3. Evaluation criteria for skin irritation intensity.

Integral mean	Stimulus intensity
0-0.49	no irritation
0.50-2.99	mild irritation
3.00-5.99	moderate irritation
6.00-8.00	severe irritation

note：Integral mean = total score of erythematous edema / number of animals in each group
